# The Laparoscopic Cholecystectomy and Common Bile Duct Exploration: A Single-Step Treatment of Pediatric Cholelithiasis and Choledocholithiasis

**DOI:** 10.3390/children9101583

**Published:** 2022-10-19

**Authors:** Zenon Pogorelić, Marko Lovrić, Miro Jukić, Zdravko Perko

**Affiliations:** 1 Department of Pediatric Surgery, University Hospital of Split, Spinčićeva 1, 21 000 Split, Croatia; 2 Department of Surgery, School of Medicine, University of Split, Šoltanska 2, 21 000 Split, Croatia; 3 Department of Surgery, University Hospital of Split, Spinčićeva 1, 21 000 Split, Croatia

**Keywords:** choledocholithiasis, cholelithiasis, children, common bile duct exploration, minimally invasive surgery

## Abstract

Background: In recent years, complicated biliary tract diseases are increasingly diagnosed in children. Laparoscopic exploration of the common bile duct (LCBDE) followed by laparoscopic cholecystectomy has gained popularity in children. The aim of this study was to investigate the outcomes of LCBDE in children and compare them with the treatment outcomes of previously used endoscopic retrograde cholangiopancreatography (ERCP). Methods: From January 2000 to January 2022, a total of 84 children (78.5% female) underwent laparoscopic cholecystectomy with a median follow-up of 11.4 (IQR 8, 14) years. Of these, 6 children underwent laparoscopic cholecystectomy (LC) + ERCP and 14 children underwent LCBDE for choledochiothiasis. The primary end point of the study was the success of treatment in terms of the incidence of complications, recurrence rate, and rate of reoperation. Secondary endpoints were stone characteristics, presenting symptoms, duration of surgery, and length of hospital stay. Results: The majority of patients were female in both groups (83.5% vs. 85.7%), mostly overweight with a median BMI of 27.9 kg/m^2^ and 27.4 kg/m^2^, respectively. Obstructive jaundice, colicky pain, acute pancreatitis, and obstruction of the papilla were the most common symptoms in both groups. The majority of patients (68%) had one stone, whereas two or more stones were found in 32% of patients. The median diameter of the common bile duct was 9 mm in both groups. The procedure was successfully completed in all patients in the ERCP group. In the group of patients treated with LCBDE, endoscopic extraction of the stone with a Dormia basket was successfully performed in ten patients (71.4%), while in the remaining four patients (28.6%) the stones were fragmented with a laser because extraction with the Dormia basket was not possible. The median operative time was 79 min in the LCBDE group (IQR 68, 98), while it was slightly longer in the ERCP group, 85 min (IQR 74, 105) (*p* = 0.125). The length of hospital stay was significantly shorter in the LCBDE group (2 vs. 4 days, *p* = 0.011). No complications occurred in the LCBDE group, while two (40%) complications occurred in the ERCP group: pancreatitis and cholangitis (*p* = 0.078). During the follow-up period, no conversions, papillotomies, or recurrences were recorded in either group. Conclusions: Exploration of the common bile duct and removal of stones by LCBDE is safe and feasible in pediatric patients for the treatment of choledocholithiasis. Through this procedure, choledocholithiasis and cholelithiasis can be treated in a single procedure without papillotomy or fluoroscopy. Compared with LC + ERCP, LCBDE is associated with a shorter hospital stay. The incidence of complications was rather low but not statistically significant.

## 1. Introduction

Cholelithiasis is the most common pathology of the biliary system. The incidence of cholelithiasis in the pediatric population has increased over the past 20 years [1,2,3,4,5]. In the past, the most common causes were related to hemolytic diseases such as hereditary spherocytosis or non-hemolytic diseases such as total parenteral nutrition, ileal diseases, or congenital biliary diseases [2,3,4]. The most common cause of gallbladder disease in the pediatric population today is obesity [3,4,5]. In the past, pigmented gallstones formed due to hemolysis of hemoglobin and its breakdown to bilirubin, which combines with calcium to form pigmented gallstones.

Nowadays, the supersaturation of bile due to excess cholesterol leads to the formation of cholesterol stones [4]. The majority of patients with gallstones are asymptomatic (80%) with an annual 1–2% risk of developing complications [5]. Usually, symptomatic patients present with abdominal pain in the right upper quadrant, often radiating to the right shoulder, nausea, and vomiting [5,6]. Complications occur as the first sign of cholelithiasis in approximately 25% of patients with gallstones [5,7]. The most common complications are biliary dyskinesia, pancreatitis, choledocholithiasis, and acute calculous cholecystitis.

Choledocholithiasis is the presence of one or more gallstones in the common bile duct. Choledocholithiasis is present in 20% of patients with cholelithiasis [8]. Depending on the origin of the stone, we can classify choledocholithiasis as primary or secondary. Primary choledocholithiasis refers to less common stones that form de novo in the biliary tree, while secondary choledocholithiasis refers to stones formed in the gallbladder [9]. Most patients with choledocholithiasis are asymptomatic. Signs of choledocholithiasis include jaundice, acute cholalangitis, pancreatitis, or sepsis. Diagnosis of choledocholithiasis includes blood tests and imaging studies. Elevated liver function tests have positive predictive value. In most patients, serum bilirubin, alkaline phosphatase, and gamma-glutamyltransferase are elevated, which has the highest sensitivity and specificity for the diagnosis of common bile duct stones [10].

Transabdominal ultrasound is the most commonly used imaging test. When the dilatation of the common bile duct exceeds 6 mm, there is a high prevalence of choledocholithiasis [11]. Magnetic resonance cholangiopancreatography (MRCP) is highly sensitive and specific in detecting choledocholithiasis. The efficiency of MRCP is comparable to invasive procedures such as endoscopic retrograde cholangiopancreatography (ERCP) and intraoperative cholangiography (IOC) [12]. ERCP has both diagnostic and therapeutic functions, but it can lead to pancreatitis, cholangitis, perforation of organs, or bleeding [13]. ERCP has the advantage of providing a direct therapeutic option when a stone is identified in the common bile duct. Treatment outcomes may depend significantly on the institution/physician performing ERCP in children (e.g., pediatric or adult gastroenterologist, high-volume center or not).

The most effective method for treating choledocholithiasis in pediatric patients remains unknown. ERCP and endoscopic sphincterotomy followed by laparoscopic cholecystectomy are the standard treatment for stones of the common bile duct in adults. Due to the risk of malignancy and higher complication rate after ERCP in the pediatric population, ERCP remains only one of the treatment options. Open cholecystectomy has been replaced by laparoscopic cholecystectomy and intraoperative cholangiography with or without exploration of the common bile duct [14]. The laparoscopic approach allows choledocholithiasis to be treated in a single procedure without papillotomy or fluoroscopy. If these approaches are unsuccessful, ERCP with endoscopic sphincterotomy can be performed, but it requires a second general anesthesia and surgical procedure [15]. The aim of this study was to evaluate the outcomes of laparoscopic exploration of the common bile duct (LCBDE) in children.

## 2. Materials and Methods

### 2.1. Patients

The case files and electronic records of 84 children (78.5% female) who underwent laparoscopic cholecystectomy in a Department of Pediatric surgery, University Hospital of Split, between 1 January 2000, and 1 January 2022, were retrospectively reviewed. Of these, a total of 14 children underwent LCBDE and in 6 children laparoscopic cholecystectomy (LC + ERCP) was performed due to choledocholithiasis and were included in further analysis. The inclusion criteria were pediatric patients who underwent ERCP or LCBDE for choledocholithiasis at our institution. The exclusion criteria were patients older than 17 years, patients who underwent conversion to open surgery, and patients with insufficient data for analysis. Patients were divided into two groups. The first group included patients in whom ERCP before LC was performed (LC + ERCP), while the second group consisted of patients in whom LCBDE was performed. ERCP was used until 2007, while only LCBDE was used thereafter.

### 2.2. Outcomes of the Study

The primary end point of the study was the success of treatment in terms of the incidence of complications, recurrence rate, and rate of reoperation. Secondary endpoints were stone characteristics, presenting symptoms, duration of surgery, and length of hospital stay.

### 2.3. Study Design

After approval by our hospital’s Institutional Review Board (IRB) (IRB reference, 500-03-/22-01/156; date of approval 25 August 2022), a retrospective cross-sectional cohort study was conducted. Case files and electronic records of patients who underwent elective laparoscopic cholecystectomy for cholelithiasis at our institution served as the data source. The patients who underwent transcystic LCBDE and stone extraction or LC + ERCP for choledocholithiasis were selected as special subgroups and included in further analysis. Demographic data, including age, sex, height, body weight, and body mass index (BMI), were recorded for each patient. The following clinical data were analyzed: presenting symptoms, duration of symptoms, comorbidities, and indications for surgery. Medical history was obtained, including concomitant diseases and the presence of hemolytic anemia. Data from imaging analysis (number and diameter of stones, diameter of common bile duct), laboratory values (total serum bilirubin, alkaline phosphatase (ALP), alanine transaminase (ALT), aspartate transaminase (AST), and gamma-glutamyl transferase (GGT)), surgical findings, surgical time, postoperative follow-up, length of hospital stay, and complications of treatment were also recorded.

### 2.4. Diagnostic Procedure and Indications for Surgery

A complete physical examination, standard laboratory tests, and ultrasonography of the abdomen were performed in all patients. In the patients with obstructive jaundice, MRCP was performed to confirm or exclude choledocholithiasis. The indication for laparoscopic cholecystectomy was symptomatic cholelithiasis and was defined as the presence of gallstones associated with recurrent biliary colic, acute cholecystitis, acute pancreatitis, obstructive jaundice (choledocholithiasis), or hemolytic anemia. An indication for treatment (LC + ERCP or LCBDE) was choledocholithiasis confirmed by MRCP.

### 2.5. Surgical Technique (LCBDE)

**Figure 1:**
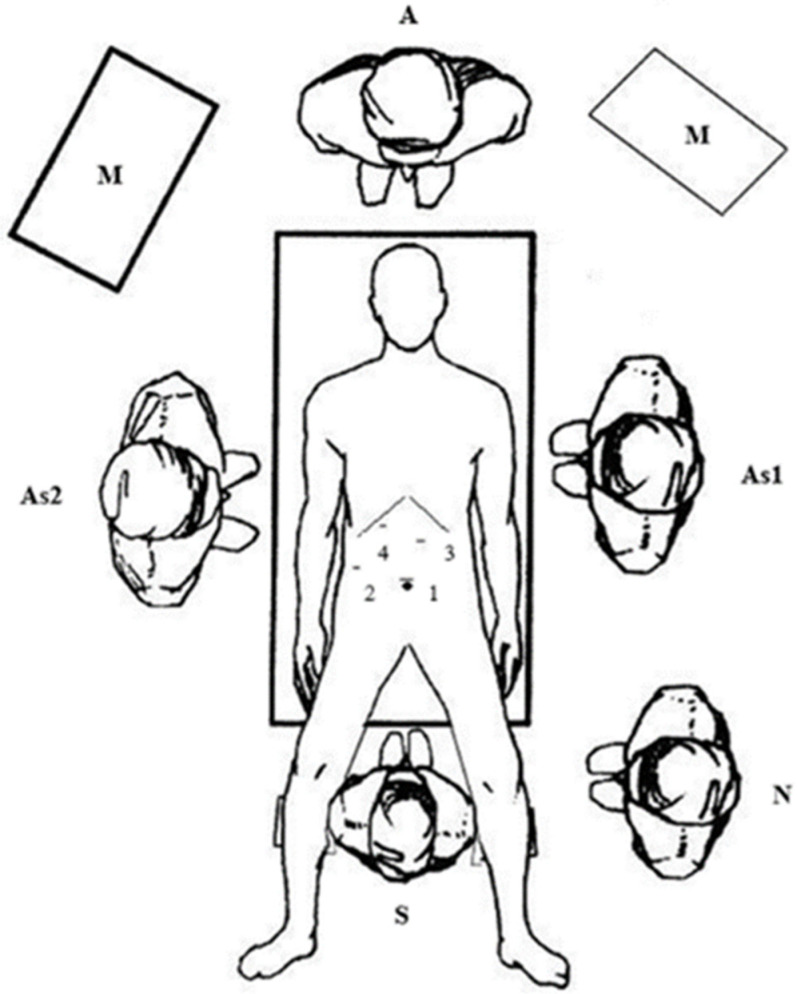
Trocar placement, position of the patient and operating staff. Legend: 1—the first 10-mm trocar; 2—the second 5-mm trocar; 3—the third (working) 10-mm trocar; 4—the fourth 5-mm trocar; S—operating surgeon; As1—the first assistant; As2—the second assistant; N—scrub nurse; A—anesthesia; M—monitor.

**Figure 2:**
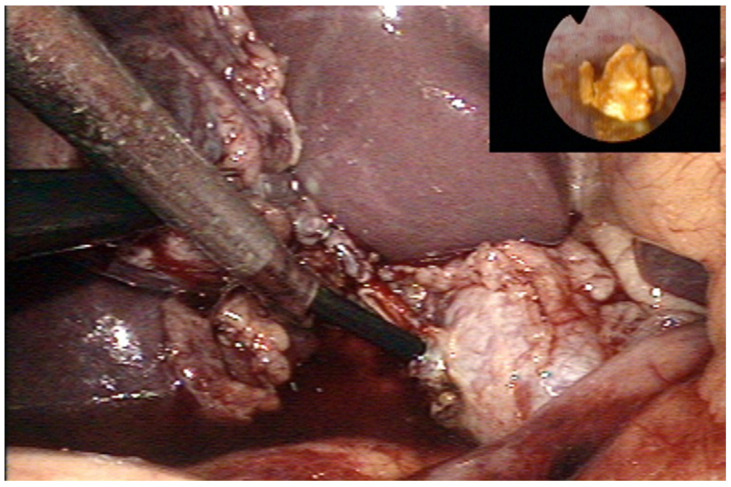
Intraoperative finding—Transcystic choledohoscopy with visualisation of the stone.

All children were treated under general anesthesia in the supine position. The surgeon was between the patient’s legs, and the assistants were on opposite sides of the patient. A surgical nurse was located on the surgeon’s right side. Two laparoscopic monitors were used, positioned next to the patient’s right and left shoulders. The trocars were positioned for laparoscopic cholecystectomy: The first 10-mm trocar for the laparoscope was placed supraumbilically, the second 5-mm trocar for the grasper was placed on the right mid-clavicular line, and the third 10-mm working trocar was placed on the left mid-clavicular line. The fourth 5-mm trocar for the choledochoscope was placed in the right subcostal position (Figure 1). After dissection of the cystic duct and artery, the cystic artery was clipped and transected. Another clip was placed at the distal part of a cystic duct, where a T-shaped incision was made. A flexible choledochoscope (CHF type CB30S; Olympus; Tokyo, Japan) was inserted through this incision, and the stone was visualized (Figure 2). After stone visualization, stones were removed using a Nitinol Tipless Dormia basket (N-Circle^®^ Nitinol Tipless Stone Extractor Cook; Bloomington, IN, USA). In cases where extraction with the Dormia basket was not possible, stones were fragmented using laser (Calculase II 20W Holmium Laser; Karl Storz; Tuttlingen, Germany) and then extracted with the Dormia basket. After successful stone extraction, the cystic duct was clamped and laparoscopic cholecystectomy was terminated in the usual manner. The abdomen was irrigated with normal saline, and the cystic stump was examined for leaks and the gallbladder bed for any bleeding before all trocars were withdrawn. The specimen was retrieved through a 10-mm trocar using an endoscopic retrieval bag (Ecosac EMP 70; Espiner Medical Ltd.; Measham, UK). All procedures were performed by two surgeons (Z. Po. and Z. Pe) with advance experience in laparoscopic surgery.

### 2.6. Follow-Up

All patients were kept in the hospital after surgery. Postoperative therapy did not differ from that for simple laparoscopic cholecystectomies; it consisted of parenteral rehydration on the first postoperative day (with enteral liquid nutrition in the evening of the day of surgery), pantoprazole, and analgesics. Antibiotics were not routinely administered. Discharge criteria included fever and good general condition, adequate postoperative oral intake, and pain control. After discharge, patients were followed up in our outpatient clinic seven days after surgery for control and suture removal and after one month to determine possible late effects.

### 2.7. Statistical Analysis

Data were analyzed using Statistical Package for Social Sciences software, version 19.0 (IBM SPSS Corp., Armonk, NY, USA). Distributions of quantitative data were described by medians and interquartile ranges (IQR), whereas categorical variables were expressed in absolute numbers and percentages. Comparative analyses were performed using the Mann-Whitney U test for continuous variables. Fisher’s exact test was used to assess differences in the distribution of categorical data.

## 3. Results

During a selected study period, a total of 84 children (78.5% females) underwent laparoscopic cholecystectomy with a median follow-up of 11.4 (IQR 8, 14) years. The demographic and clinical characteristics of the children who underwent cholecystectomy during the study period are shown in Table 1.

Of the total number of children who underwent laparoscopic cholecystectomy, 6 (%) children underwent LC + ERCP and 14 children underwent LCBDE, due to choledochothiasis. Of the patients who underwent LC + ERCP or LCBDE, complete data were available for all patients included in analysis. The majority of patients were female in both groups (83.5% vs. 85.7%), mostly overweight with a median BMI of 27.9 kg/m^2^ and 27.4 kg/m^2^, respectively.

Obstructive jaundice, colicky pain, acute pancreatitis, and obstruction of the papilla were the most common symptoms in both groups. The majority of patients (68%) had one stone, whereas two or more stones were found in 32% of patients. The median diameter of the CBD was 9 mm in both groups. The procedure was successfully completed in all patients in the ERCP group. In the group of patients treated with LCBDE, endoscopic extraction of the stone with a Dormia basket was successfully performed in ten patients (71.4%), while in the remaining four patients (28.6%) the stones were crushed with the laser because extraction with the Dormia basket was not possible.

The median operative time in the LCBDE group was 79 min (IQR 68, 98), whereas it was slightly longer in the ERCP group, 85 min (IQR 74, 105), but with no statistically significant difference (*p* = 0.125). The length of hospital stay was significantly shorter in the LCBDE group (2 vs. 4 days, *p* = 0.011). No complications occurred in the LCBDE group, while two (40%) complications occurred in the ERCP group, pancreatitis and cholangitis (*p* = 0.078).

During the follow-up period, no conversions, papillotomies, or recurrences were recorded in either group. The demographic and clinical characteristics of the children who underwent ERCP and LCBDE during the study period are shown in Table 2.

The spectrophotometric analysis of gallstones and the histopathologic report of children who underwent LCBDE during the study period are shown in Table 3. The spectrophotometric analysis data for children who underwent ERCP were not available.

## 4. Discussion

This study clearly demonstrates that LCBDE is a safe and effective surgical procedure in pediatric patients with choledocholithiasis. The efficacy of this method was demonstrated by the fact that all of our patients who underwent LCBDE recovered from choledocholithiasis without recurrence or postoperative complications. Most patients in our study group were female and had a higher body mass index. They presented with colicky pain, obstructive jaundice, and elevated liver enzymes due to obstruction, acute pancreatitis, or obstruction of the papilla. Compared with LC + ERCP, LCBDE is associated with a shorter hospital stay. The incidence of complications was rather low but not statistically significant, probably due to the small sample size.

The incidence of cholelithiasis and its complications, including choledocholithiasis, in the pediatric population has increased significantly over the past two decades [1,2,3,4,5,16]. The causes are multifactorial, and the main risk factor for choledocholithiasis is obesity [5,17]. In addition, the increasing use of abdominal ultrasonography has led to better detection of asymptomatic stones of the common bile duct [18]. In the treatment of pediatric choledocholithiasis, the optimal approach remains unclear. Therapeutic techniques include laparotomy, laparoscopic surgery, percutaneous intervention, and endoscopic retrograde cholangiopancreatography (ERCP) [19].

The approach most commonly used in adult patients involves a two-stage procedure: preoperative ERCP with stone extraction, followed by laparoscopic cholecystectomy. Laparoscopic cholecystectomy is usually performed after ERCP to reduce recurrent biliary events [20]. ERCP in the pediatric population can lead to numerous adverse events. The most common complication is ERCP-induced pancreatitis [21,22]. Most cases of pancreatitis after ERCP are usually mild and do not require surgery [22]. Other complications include abdominal pain after the procedure, gastrointestinal bleeding after sphincterotomy, perforation, and cholangitis. In addition, if impacted, large, or multiple stones are present in the common bile duct, it may be problematic to remove them with ERCP [23,24]. A single-stage procedure is an open or, more commonly, an LCBDE. LCBDE is both a diagnostic and therapeutic procedure that can be performed via the cystic duct or an incision of the common bile duct. Transcystic stone extraction is preferred when the number of stones is smaller (up to 5 stones), the size of the stones is smaller (up to 9 mm), and the width of the common bile duct is 15 mm or less. In other cases, laparoscopic choledochotomy is recommended. The efficiency of transcystic stone extraction is 85–95% [24]. If choledochoscopy is performed via a choledochothomy incision, the efficiency increases up to 100% [24].

Bansal et al. showed that patients who underwent LCBDE had a longer mean operative time than patients who had ERCP with laparoscopic cholecystectomy (135.7 ± 36.6 min vs. 72.4 ± 27.6 min; *p* ≤ 0.001), but they had a significantly shorter hospital stay (4.6 ± 2.4 days vs. 5.3 ± 6.2 days; *p* = 0.03) [25]. Kim et al. reported a mean operative time of 92.0 ± 38.6 min and a mean hospital stay of 7.4 ± 3.7 days [19]. In our study, the median of operative time was 79 min (IQR 68, 98) and the median length of hospital stay was 2 days (IQR 2, 4) in the LCBDE group, whereas it was significantly longer (4 days) in the group of patients treated with LC + ERCP.

In their study of 42 pediatric patients, Short et al. showed that LCBDE patients had no major complications, whereas LC + ERCP patients had two major complications (one patient with duodenal perforation and one patient with bleeding requiring transfusion). In our study, a similar distribution was found: no major complications in the LCBDE group, while two complications (cholangitis and pancreatitis) were noted in the LC + ERCP group. They came to similar conclusions as in our study: the LCBDE is associated with shorter hospital stay and lower costs, and has similar or better morbidity than the LC + ERCP [26].

A randomized control trial compared complication rates in patients undergoing a one-stage procedure with those undergoing the two-stage procedure. Superficial surgical site infection occurred in 7.1% of patients in both groups. In addition, bile leakage was noted in 16.7% of patients [25]. Perko et al. reported excellent results of LCBDE in a cohort of thirteen adult patients. They concluded that this method is safe and effective for the treatment of choledocholithiasis and that open surgery and ERCP/sphincterotomy should be avoided in patients with choledocholithiasis who require laparoscopic cholecystectomy [24].

During our follow-up period, none of the patients experienced postoperative complications or recurrences. The majority of pediatric patients with choledocholithiasis are 12–15 year old females (72.6–77.4%) with an average weight of 58.3 kg [27,28,29]. In our study group, the mean age was 13.5 years (IQR 11–14.5), with 85.7% female patients whose BMI was above average. Due to excess cholesterol, most patients in recent studies had cholesterol gallstones [3,5,30]. Chamorro et al. reported that 86.6% of their patients had cholesterol stones [30]. Walker et al. also reported a predominance of cholesterol stones. Non-hemolytic cholesterol stones and biliary dyskinesia were associated with patients with higher BMI [3]. In our study group, there were 50% patients with cholesterol gallstones and 7.1% with mixed gallstones.

**Figure 3:**
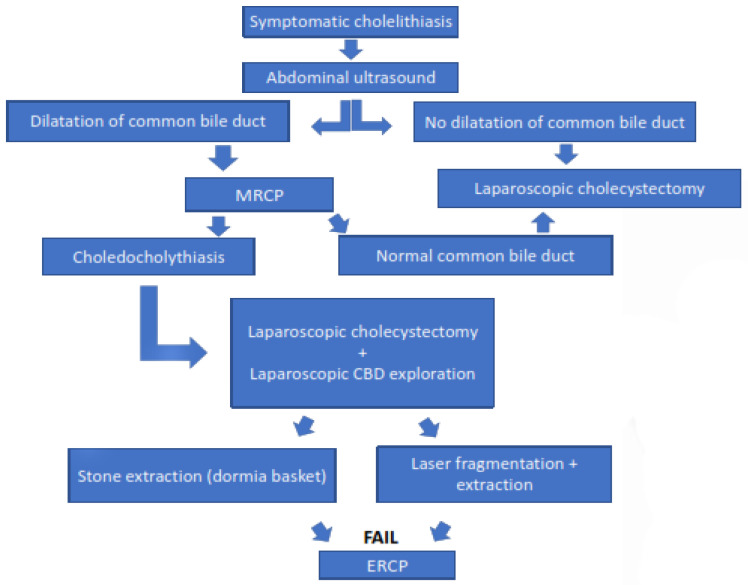
Algorithm of treatment of the pediatric patients with choledocholithiasis.

To date, numerous strategies and algorithms have been published for the optimal treatment of choledocholithiasis. Based on our experience and this 20-year retrospective study, we have developed an algorithm for pediatric patients with gallstones (Figure 3). All pediatric patients with symptomatic cholelithiasis should undergo abdominal ultrasonography. If there is no dilatation of the common bile duct and there is no clinical evidence of choledocholithiasis, the patient with symptomatic cholelithiasis is scheduled for elective laparoscopic cholecystectomy. If there is dilatation of the common bile duct on abdominal ultrasound and clinical signs of choledocholithiasis, magnetic resonance cholangiopancreatography (MRCP) should be performed. If MRCP detects stones in the common bile duct, the patient should undergo a single-stage LCBDE that includes laparoscopic cholecystectomy and laparoscopic exploration of the common bile duct. After exposure of the stone, the stone should be removed with the Dormia basket. If removal of the stone with the Dormia basket is not possible, it can be crushed with a laser and then removed with the Dormia basket. Only in cases where LCBDE cannot remove the stone should ERCP be performed for stone removal.

### Limitations of Study

This study has limitations because of its retrospective nature and relatively small number of patients, as it is a single-center study. In addition, because of the low frequency of choledocholithiasis in the pediatric population, we may not have the most effective algorithm. A multicenter study design based on a larger patient population would provide us with more valid results and lead to more efficient treatment of patients with choledocholithiasis.

## 5. Conclusions

Exploration of the common bile duct and removal of stones by LCBDE is safe and feasible in pediatric patients for the treatment of choledocholithiasis. Through this procedure, choledocholithiasis and cholelithiasis can be treated in a single procedure without papillotomy or fluoroscopy. Compared with ERCP, LCBDE is associated with a shorter hospital stay. The incidence of complications was rather low but not statistically significant.

## Figures and Tables

**Table 1:** Demographic and clinical characteristics of children who underwent cholecystectomy during the study period.

Variable	2000–2022
Number of cholecystectomies	84
Demographic data	
age; years (median, IQR)	11.4 (8, 14)
female; *n* (%)	66 (78.5%)
male; *n* (%)	18 (21.5%)
Anthropometric data	
body height; cm (median, IQR)	147.5 (138.8, 154.9)
body mass; kg (median, IQR)	55.9 (50.3, 63.8)
BMI; cm/m^2^ (median, IQR)	26.7 (22.4, 29.6)
Main indication for surgery	
biliary colic; *n* (%)	14 (16.7%)
hereditary spherocytosis; *n* (%)	15 (17.9%)
acute pancreatitis; *n* (%)	16 (19%)
acute cholecystitis; *n* (%)	19 (22.6%)
obstructive jaundice; *n* (%)	20 (23.8%)
Complications; *n* (%)	
CBD injury	1 (1.3%)

BMI—Body Mass Index; IQR—Inter Quartile Range; CBD—Common bile duct.

**Table 2:** Demographic and clinical characteristics of children who underwent LCBDE.

Variable	LC + ERCP (2000–2007)	LCBDE (2007–2022)	*p*
Number of procedures—LCBDE; *n* (%)	6 (30)	14 (70)	<0.001
Demographic data Gender—female; *n* (%) Age; years (median, IQR) BMI; cm/m^2^ (median, IQR)	5 (83.5) 14 (11.5, 145) 27.9 (23.5, 29.5)	12 (85.7) 13.5 (11, 14.5) 27.4 (23, 29.6)	0.891 0.651 0.722
Clinical presentation; *n* (%)			
obstructive jaundice colic type pain acute pancreatitis obstruction of papilla Laboratory values (median, IQR) Bilirubin, μmol/L ALP, U/L AST, U/L ALT, U/L GGT, U/L	6 (100) 6 (100) 2 (60) 2 (40) 129 (91, 204) 1174 (1051, 1387) 133 (112, 159) 196 (173, 211) 186 (169, 201)	14 (100) 13 (92.9) 7 (50) 5 (41.6) 132 (89, 206) 1105 (1004, 1354) 134 (114, 158) 188 (169, 206) 186 (171, 200)	1.0 0.874 0.642 0.918 0.874 0.541 0.698 0.884
Stone characteristics Diameter; mm (median, IQR) Number of stones (median, IQR) Diameter of CBD; mm (median, IQR) Surgical approach; *n* (%) LCBDE—Dormia basket extraction LCBDE—Laser fragmentation	8 (6.5, 11) 1 (1, 2) 9 (8, 11) - -	7.5 (6, 11) 1 (1, 3) 9 (7.5, 11) 10 (78.5) 4 (21.5)	0.478 0.908 0.854
Complications; *n* (%)	2 (40)	0 (0)	0.078
Operative time; min (median, IQR) LOS; days (median, IQR)	85 (74, 105) 4 (3, 5)	79 (68, 98) 2 (2, 4)	0.125 0.011

LC—Laparoscopic Cholecystectomy; ERCP—Endoscopic Retrograde Cholangiopancreatography; LCBDE—Laparoscopic common bile duct exploration; BMI—Body Mass Index; IQR—Inter Quartile Range; ALP—alkaline phosphatase, ALT—alanine transaminase, AST—aspartate aminotransferase, GGT—gamma-glutamyl tansferase; CBD—Common bile duct; LOS—Length of Hospital Stay.

**Table 3:** Spectrophotometric analysis of gallstones and histopathology report of children who underwent LCBDE.

Variable	*n* (%)
Type of gallstone	
pigment	6 (42.9)
cholesterol	7 (50)
mixed	1 (7.1)
Histopathology report	
normal	1 (7.1)
acute cholecystitis	2 (14.3)
chronic cholecystitis	11 (78.6)

## Data Availability

The data presented in this study is available upon request of the respective author. Due to the protection of personal data, the data is not publicly available.
